# Does preventive single dose of intravenous dexketoprofen reduce pain and swelling after orthognathic surgery? A prospective, randomized, double blind clinical trial

**DOI:** 10.4317/medoral.24852

**Published:** 2023-04-26

**Authors:** Yusuf Nuri Kaba, Ahmet Emin Demirbas, Nükhet Kütük, Dilek Günay Canpolat, Alper Alkan

**Affiliations:** 1 DDS, Assistant Professor, Department of Oral and Maxillofacial Surgery, Erciyes University Faculty of Dentistry, Melikgazi, Kayseri, Turkey; 2 DDS, PhD, Assistant Professor, Department Head, Department of Oral and Maxillofacial Surgery, Erciyes University Faculty of Dentistry, Melikgazi, Kayseri, Turkey; 3 DDS, PhD, Associate Professor, Department of Oral and Maxillofacial Surgery, Bezmialem Vakif University, Faculty of Dentistry, Istanbul, Turkey; 4 MD, Associate Professor, Anaesthesiologist, Department of Oral and Maxillofacial Surgery, Erciyes University Faculty of Dentistry, Melikgazi, Kayseri, Turkey; 5 DDS, PhD, Professor, Department of Oral and Maxillofacial Surgery, Bezmialem Vakif University, Faculty of Dentistry, Istanbul, Turkey

## Abstract

**Background:**

The purpose of this study was to evaluate the effect of a single-dose intravenous dexketoprofen administration for preventive analgesia on postoperative pain and reducing swelling in double jaw surgery.

**Material and Methods:**

The authors designed a prospective, randomized, and double-blind cohort study. Patients who have Class III malocclusion were randomly divided in two groups. 50 mg intravenous dexketoprofen trometamol were administrated 30 minutes before incision in treatment group, while intravenous sterile saline was administrated 30 minutes before incision in placebo group. The primary predictor variable was treatment group. Primary outcomes were pain, swelling and 24-hour opioid intake. Patient- controlled analgesia with tramadol was given for management of postoperative pain. Other variables were demographic and operation related parameters. Visual analogue scale was used to evaluate postoperative pain. 3dMD Face System (3dMD, USA) was used to measure postoperative swelling. Data were analysed using two independent samples t test and Mann Whitney U test.

**Results:**

The study sample was composed of 30 patients with a mean age of 20,63 years and 21 were female. Preemptive dexketoprofen administration decreased postoperative tramadol consumption by 25.9% compared to placebo group, and there was a statistically significant decrease in VAS scores (*p*<0,05). There was no statistically significant difference between the groups in terms of swelling (*p*>0,05).

**Conclusions:**

Preventive administration of intravenous dexketoprofen provides adequate analgesic effect in the postoperative 24-hour period and reduces opioid consumption in orthognathic surgery.

** Key words:**Preventive analgesia, pain, swelling, orthognathic surgery, dexketoprofen, opioid, 3dMD.

## Introduction

Orthognathic surgery is invasive, major and well-established surgical operation which was widely used to correct dentofacial deformities across the globe. The main complaints of the patients after orthognathic surgery are pain, swelling, trismus, social and functional disabilities. Bimaxillary orthognathic surgery procedures are known to cause moderate to severe pain that requires the use of opioids (1). Acute pain is a challenge problem during postoperative recovery in clinics and increase patient comorbidities patient after surgery. The main reasons for hospitalization are postoperative pain and swelling after orthognathic surgery. Most of patients are hospitalized a few days for postoperative care after orthognathic surgery (2). Postoperative pain and swelling occur due to tissue injury as a result of wide mucoperiosteal flap elevation, muscle stripping, osteotomes, chisel, saw, drills and retractor usage during orthognathic surgery (3). If postoperative pain and swelling cannot be adequately controlled, recovery time, hospitalization period and patient costs increase, and patient satisfaction decreases. Post-operative pain is defined as acute pain that develops as a result of an inflammatory process caused by surgical trauma. It starts after surgical trauma and decreases and disappears with recovery. It is different from other type of acute pains due to its etiology and is therefore expected to occur almost all the time.

Preemptive analgesia is one of the most popular subjects of pain management. Preemptive analgesia was first introduced by Crile in 1913 (4). Since then, this concept has been widely recognized for the treatment of patients in almost every surgical discipline. The purpose of preventive analgesia is to prevent the perception of pain by preventing peripheral and central sensitivity due to harmful stimulation (5). The main idea of pre-emptive analgesia protocols is starting the analgesics before the surgical incision. It refers to an analgesic regimen to control sensitization due to tissue damage, preoperatively, intraoperatively or at any time the broad comprehensive definition of preventive analgesia has recently become quite popular. Preemptive and preventive analgesia relieve acute pain and thus prevent it from developing into chronic pain (6).

In order to provide postoperative analgesia in bimaxillary surgery, many drugs and methods are used such as steroids, opioids (7), nonsteroidal anti-inflammatory drugs (NSAIDs) (3,7-8) local anaesthetics (3), antiepileptics(9), antiemetics (10), regional nerve block (11) , and different cooling methods (12). Although opioids are excellent for management of severe pain, they are also known have the potential for misuse and addiction (13). The efficacy of non-steroidal anti-inflammatory drugs in reducing pain and opioid consumption after surgery is well documented in the literature. Because of decreased pulmonary and gastrointestinal side effects and provide adequate analgesia without abuse and addiction compared to opioids, the NSAIDs are widely used in postoperative analgesia (14). Dexketoprofen trometamol, the active enantiomer of racemic ketoprofen, is a NSAID with analgesic and anti-pyretic properties. An aryl propionic acid derivative, the S (+) enantiomer of ketoprofen, is considered a a strong inhibitor *in vitro* prostaglandin synthesis. Dexketoprofen trometamol has been shown to be effective in the management of acute pain (15).

The authors hypothesize that preventive administration of dexketoprofen may reduce postoperative pain and swelling in double jaw surgery. The main objective of the research was to assess the effects of preventive intravenous (iv) dexketoprofen trometamol administration on postoperative pain and swelling in orthognathic surgery.

## Material and Methods

- Study Design/Sample

This cohort study was designed by the researchers as a single-center, prospective, controlled, randomized, and double-blind study. Ethical approval for this study was obtained from the Erciyes University Human Research Ethics Committee. This study was performed on patients who had undergone double jaw surgery to correct Class III skeletal deformity between 2017 and 2019 in Erciyes University Faculty of Dentistry, Department of Oral and Maxillofacial Surgery. The number of samples was calculated using the Cohen approach to have 80% power and 0.05 error. Based on this calculation, it was decided to include 15 patients in the study. All the volunteers were informed about the drugs, surgical procedure, possible side effects and complications and written informed consent was obtained.

Inclusion criteria for this study were as follows; patients 18-45-year-olds, ASA I status, Class III malocclusion, elective double jaw surgery. Exclusion criteria were ASA II and above, drug allergy, liver and kidney failure, pregnant or breastfeeding, long-term use of pain relievers such as NSAIDs and opioids, diabetes.

- Predictor Variable

The primary predictor variable was preventive analgesia group. Patients were randomly divided in two groups. 50 mg IV dexketoprofen trometamol (Arveles 50mg/2mL; UFSA, İstanbul, Turkey) were administrated 30 minutes before incision in the treatment group (deksketoprofen trometamol *n*= 15) and IV sterile saline were administrated 30 minutes before incision in the placebo group (saline *n*= 15). Before the operation, all patients were informed about the use of patient control analgesia (PCA) device and VAS (Visual Analog Scale). In order to provide double blindness different researchers performed preventive medication and evaluation of the postoperative edema and pain.

- Other variables

Demographic and operation related parameters possibly associated with postoperative pain and swelling were recorded. Demographic variable was age, gender and weight. Operation related variable were amount of movement of upper and lower jaw, amount of bleeding, duration of surgery and vomiting.

- Primary Outcomes

The postoperative pain, swelling and 24-hour opioid intake were primary outcomes. The postoperative pain was assessed using a 10-cm visual analogue scale (VAS) at postoperative 1st, 3rd, 6th, 9th, 12th and 24th hours. Complications such as nausea, vomiting, rash, itching were also noted.

**Figure 1:**
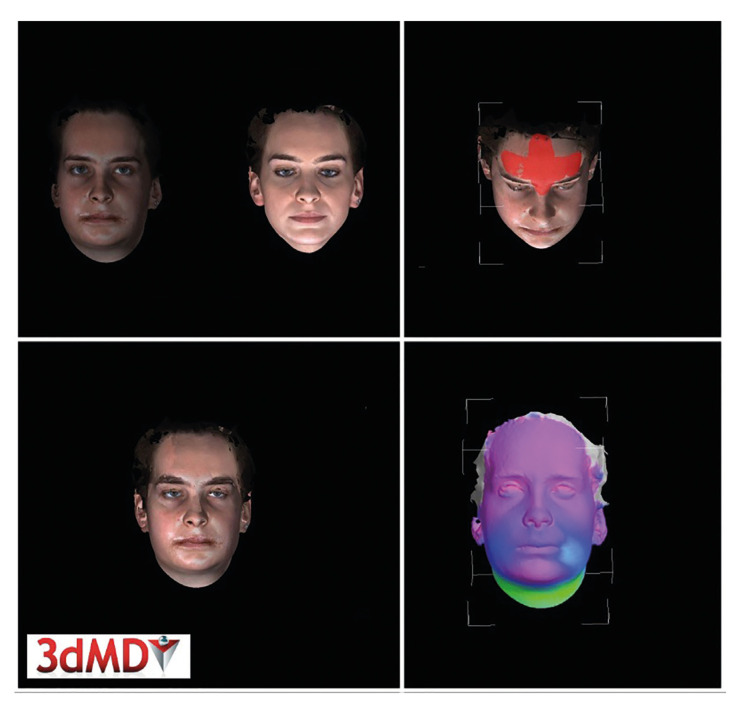
Acquisition of 3D images with 3dMD Face system.

3dMD Face System (3dMD, ATLANTA, GA, USA) was used for 3D imaging of postoperative swelling. The same clinician took the 3D images from patients in natural head position, maximum intercuspation and eyes open. 3D images were taken one (T0) day before surgery and 1 (T1), 3 (T3), 7 (T7), 14 (T14), 21 (T21), 30 (T30), 90 (T90) day after surgery. The 3dMD Vultus Software (3dMD, Atlanta, GA) was used to analyse the images. The 3-month postoperative 3D image (t90) was selected as the reference image to compare with other images. After cutting and customizing the images by anatomical structures, scans t0-t30 were matched to the reference scan t90. The forehead, nasion, tragus, medial and lateral canthus were used for surface matching. After surface matching, the volume difference between the masks was calculated (Fig. 1).

- Secondary Outcome

Secondary outcomes were rescue analgesic requirement and relationship between bleeding, duration of surgery, pain and swelling.

- Surgical Procedures

8 mg ondansetron (Ondaren of 4 mg / 2 mL, VEM, Istanbul, Turkey), 2 mg midazolam (Demizolam, Dem İlaç San. Tic. Ltd. Şti. Istanbul, Turkey), were administered to all patients as a premedication 30 minutes before the operation. For antibiotic prophylaxis iv 2 gr ampicillin + sulbactam (Alfacid Im/Iv, Yavuz İlaç Ecza Deposu, Istanbul, Turkey) was given all patient 30 minnute before operation.

The same surgical team in Erciyes University Oral and Maxillofacial Surgery Hospital operated all patients. Anesthesia induction was performed in both groups by IV 2 mg/kg propofol (Propofol®, Fresenius Kabi, Germany) and IV 0.6 mg/kg rocuronium (Esmeron®, Glaxosmithkline, UK). Patients were intubated with nasotracheal approach. 0.1 mg/kg rocuronium was given for muscle relaxation during the operation whenever necessary. Maintenance of anesthesia was provided by 1.5% sevofluran (Sevorane®, Abbott, USA) and 50 μg /h fentanyl, 50% N2O in oxygen, 50% O2 for air. (Tidal Volume= 6 8mL/kg, Frequency =10/mins). During the operation, 1μcg/kg fentanyl was made when require an additional analgesia. The inferior alveolar, buccal and lingual block were performed with 80 mg %2 articaine in addition to 1:200.000 epinephrine (Ultracain®%2 Ampoule, Sanofi Aventis, Istanbul, Turkey) to all patients before surgery. Le fort I (LFI) osteotomy and Bilateral Sagittal Split Osteotomy (BSSO) was performed according to Bell and Hunsuck modification. 1mg neostigmine (Neostigmin® Ampoule 0.5mg/mL, Adeka, Samsun, Turkey) and 0.5mg atropine (Atropine Sulphate® Ampoule 0.5mg/mL, Galen, Istanbul, Turkey) were administered for antagonism of the muscle relaxant when the surgical process was finished. Duration of surgery, total amount of intraoperative bleeding and complications were recorded. The patient extubated and transferred to the post anaesthetics care unit (PACU).

- Postoperative Care

Pressure elastic patches were applied to the patients to include lip, cheek and submandibular areas for edema control. All the patients received iv tramadol (Contramal 100 mg, Abdi İbrahim, İstanbul, Turkey) with PCA device (Abbott Pain Management Provider, Chicago, IL) at postoperative care unit. The PCA device was adjusted to have a loading dose of 50 mg, a bolus dose of 10 mg, a 30-minute lock-in time, no basal infusion, and a 4-hour restriction. When the VAS pain score is 4 and/or higher than 4, rescue analgesia was provided by iv 1000 mg paracetamol (Perfalgan 10 mg/mL 100 mL, Bristol-Myers Squibb, İstanbul, Turkey)

- Data Analyses

The normal distribution of the data was evaluated by histogram, q-q graphs and Shapiro-Wilk test. The homogeneity of variance was tested by the Levene test. In the comparisons between the two groups, two independent samples t test and Mann Whitney U tests were used for the quantitative variables. The relationship between quantitative variables was evaluated by Spearman correlation analysis. Pearson m2 analysis was used to compare categorical data. The Friedman test was used for time comparisons. Dunn test was used for multiple comparisons. Data analysis TURCOSA Statistics Software (Turcosa Analytical Ltd. Co., is www.turcosa.com.t Turkey) was held at the statistics software. Significance level was accepted as *p* <0.05.

## Results

- Demographic Results

A total of 30 patients, 6 males/9 females in Group I and 3 males/12 females in Group II, were included in the study. The mean age was 21.07±2.22 in Group I and 20.20±3.73 in Group II. There was no statistically significant difference between the groups in terms of age, sex and weight (*p*> 0,05). Comparison of surgical planning, duration of surgery, intraoperative bleeding, and postoperative vomiting between two groups were summarized in Table 1. There was no statistically significant difference between the groups in terms of maxillary advancement, maxillary impaction, mandibular setback, total activation, duration of surgery, intraoperative bleeding, and postoperative vomiting ​​(p> 0.05).

- Primary Outcome

**Figure 2:**
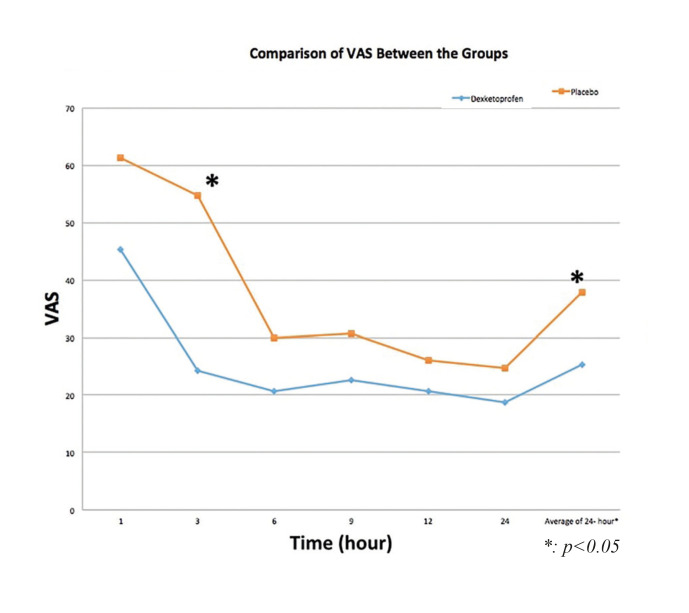
Pain intensity (VAS values) means during 24 h after surgery.

Pain:

Comparison of mean VAS values between the groups during the first 24 hour postoperative period were described in Table 2. The mean values of postoperative 24-hour VAS were significantly lower in the study group compared to the placebo group (*p* = 0.013). There was no statistically significant difference between the groups in terms of VAS except the mean values of VAS postoperative 3rd hour ​​and postoperative 24-hour VAS mean values (Fig. 2).

**Table 1:**
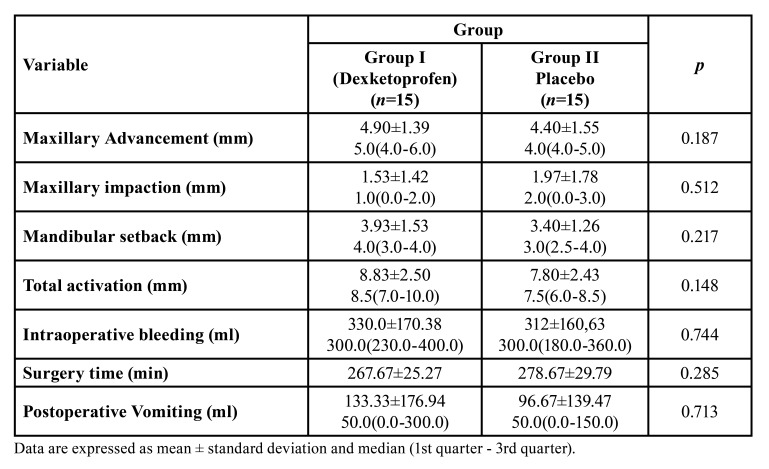
Comparison of operation related variable and vomiting.

**Table 2:**
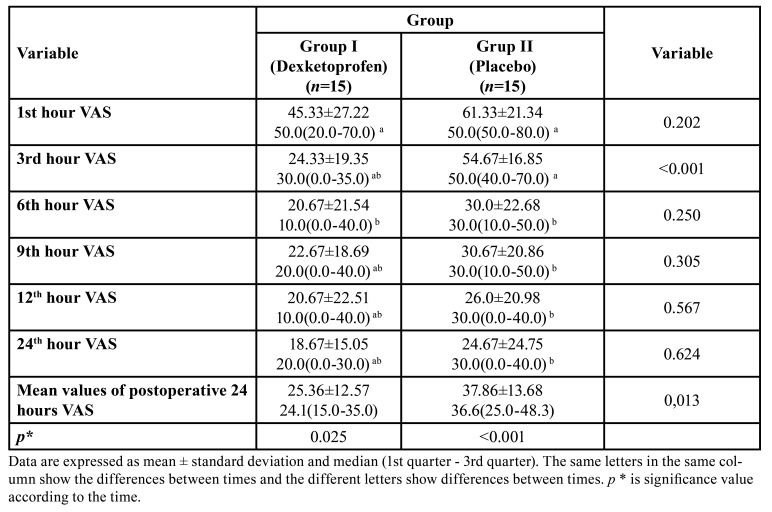
Comparison of VAS between the groups during the 24-hour postoperative period.

Postoperative 3rd hour VAS mean values ​​were significantly lower in the study group compared to the placebo group (*p* <0.001). There was a statistically significant difference between the 1st hour and 3rd hour in study group (dexketoprofen) in the comparison of mean VAS values according to the time (*p* = 0.025). In placebo group, there was a statistically significant difference between the 1st hour with 3rd hour and 6th hour (*p* <0.001). There was also a statistically significant correlation between postoperative 3rd hour mean VAS values and 24-hour mean VAS values (*p* <0.01).

Comparison of tramadol consumption of postoperative 24 hours and requirements of rescue analgesic between the two groups were summarized in Table 3. Tramadol consumption of postoperative 24 hours was 109.90±35.54 in the study group, while 148.17±45.13 in the placebo group.

**Table 3:**
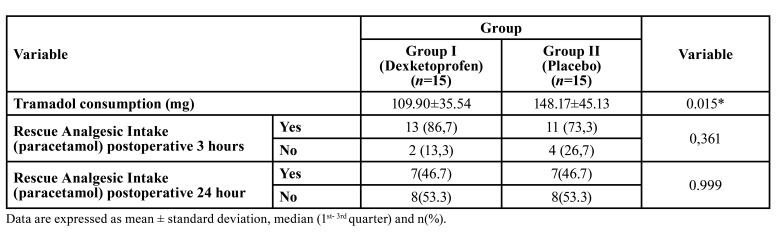
Comparison of tramadol consumption of postoperative 24-hours and requirements of rescue analgesic between the two groups.

**Figure 3:**
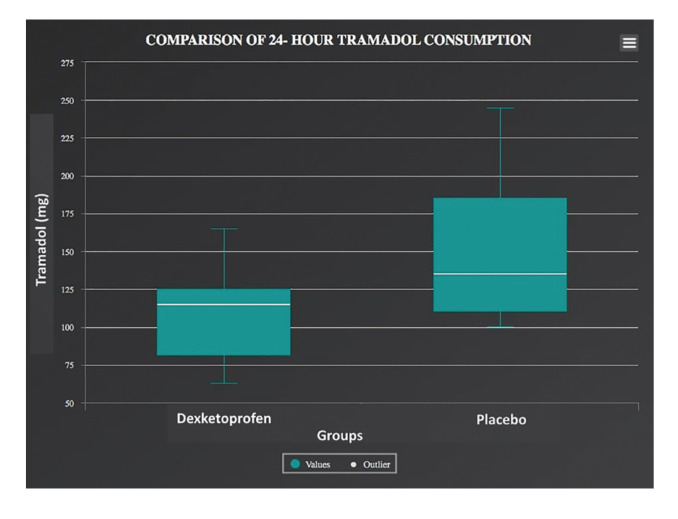
Analysis of the average tramadol intake during the 24 h period after surgery.

There was a statistically significant difference between the groups in terms of opioid consumption (*p*=0,015) (Fig. 3).

**Figure 4:**
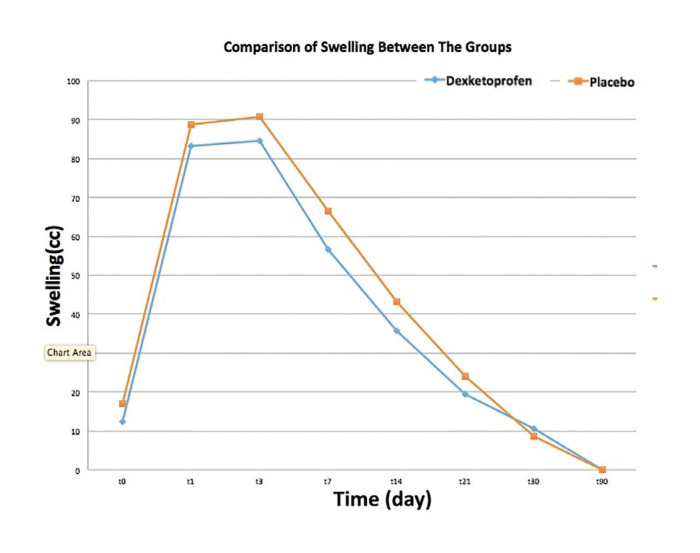
Comparison of Swelling between the groups.

Swelling:

Comparison of mean values of postoperative swelling between the groups was showed in Table 4. There was no statistically significant difference between the groups in terms of postoperative swelling (*p* <0.01) (Fig. 4).

Secondary Outcome:

There is no statistical difference between the groups in terms of rescue analgesic requirements (*p*=0,999). In addition, there was a moderate and positive correlation between maxillary impaction and postoperative 24-hour tramadol consumption (*p* <0.01).

**Table 4:**
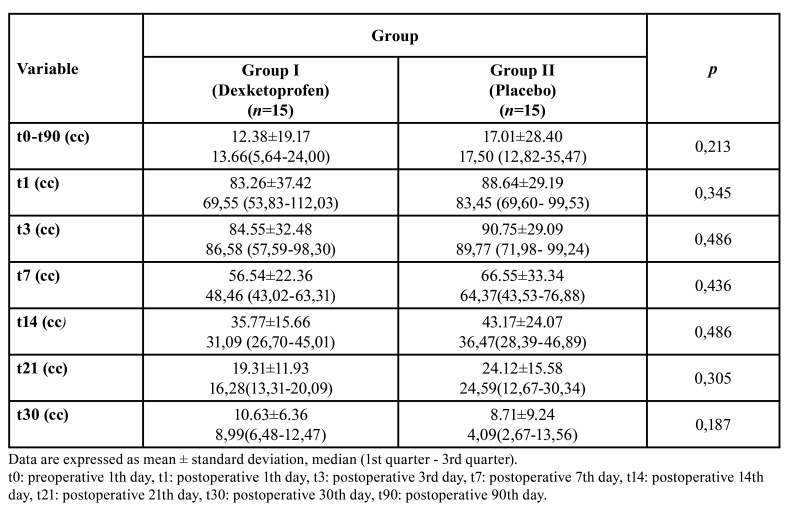
Comparison mean values of postoperative swelling between the groups.

## Discussion

The aim of this study was to evaluate the effectiveness of preventive IV dexketoprofen on postoperative swelling and pain in patients after orthognathic surgery. In recent years, significant advances in anesthesia techniques have emerged to reduce postoperative pain. Bimaxillary surgery which includes BSSO and LF I osteotomy is well documented orthognathic surgical procedure. Postoperative pain, swelling, inferior alveolar nerve paraesthesia, difference between patient expectations and operation outcome, and postoperative speech problems were shown as sign of low satisfaction after orthognathic surgery (16) Niederhagen *et al*.(17) reported that double jaw surgeries are the highest painful procedures in maxillofacial surgical practice. The ideal postoperative pain management provide adequate analgesia while reducing dose and side effects. However, a standard treatment modality and drug for each patient was not found. If pain therapy is initiated after a surgical stimulus; peripheral and central system sensitization system may develop and postoperative pain treatment may be difficult. Multimodal analgesia is the preferred method for managing pain caused by peripheral and central sensitization. The aim of preemptive analgesia is to reduce postoperative pain by preventing peripheral and central sensitization (5). Management of postoperative pain is more important nowadays in preoperative, intraoperative and postoperative period. Thus, both preventive and preventive analgesia can be administered to centrally reduce and prevent modulation of acute pain. Thus, the transformation of acute pain into chronic pain is prevented (5). This study was also carried out in a multimodal analgesia approach that started with a single protective dose of dexketoprofen and continued with tramadol with PCA and paracetamol for resque analgesic in the postoperative 24-hour period.

Many systemic agents were used preoperatively to provide adequate analgesia in orthognathic surgery. Naproxen, diclofenac, tramadol, pregabalin, rofecoxib, ondansetron and clonidine are among the drugs used for this purpose(3,7,9-10,18). Preoperative regional nerve block was applied to reduce postoperative pain also (11). There are currently no accepted or approved optimal pain treatment protocol to reduce postoperative pain in double jaw surgery. The authors hypothesize that preventive administration of dexketoprofen may reduce postoperative pain, swelling and opioid intake in double jaw surgery. Ketoprofen is an agent with analgesic and anti-inflammatory activity. It is one of the most potent *in vitro* inhibitors of prostaglandin synthesis, but is also thought to be associated with a higher risk of serious gastrointestinal bleeding than other NSAIDs(19). Dexketoprofen trometamol is the water-soluble S (+) enantiomer of ketoprofen. Because it is more lipophilic than ketoprofen and its solubility is increased by adding trometamol (36.9 mg) to the molecule, the effect of dexketoprofen starts faster and the gastrointestinal side effects are less. In this way, it is aimed to reduce the incidence of side effects while providing the same analgesic effect with lower doses. In a study conducted in Spain, it was shown that dexketoprofen exhibits a lower gastrointestinal side-effect profile than ketoprofen (19). Hanna *et al*. (20) reported that dexketoprofen trometamol has analgesic efficacy similar to COX-2 inhibitors in the treatment of acute pain, reduces opioid use when applied with a multimodal analgesia approach, has a rapid onset of action, and is well tolerated. Analgesic efficacy of preemptive dexketoprofen was well-documented in the literature. It has been reported that it reduces postoperative opioid consumption and decreases VAS scores in many different types of surgery (21). Therefore, in this study, preventive iv dexketoprofen trometamol was preferred because of its advantages such as rapid onset of action, well tolerated, and reducing opioid intake. This study was designed as a prospective, randomized, double-blind, controlled study to ensure standardization and increase the scientific impact of the study. In dexketoprofen group statistically significant decrease was observed in terms of postoperative tramadol consumption compared with placebo (*p*=0,015). Also, postoperative 3rd hour and 24 hours mean VAS values ​​were significantly lower in dexketoprofen group compared to placebo group (*p*<0.001).

The application of preventive analgesic may start days prior to surgery or just before surgical stimulation (22) . In this study, preincisionel approach was preferred in preventive analgesia. Double jaw surgery causes the most severe pain compared the other orthognathic procedures. It’s also reported that positively correlation between duration of operation and postoperative pain (7,17,23). Therefore, the study was carried out on 30 patients scheduled for double jaw surgery due to class 3 skeletal orthodontic anomalies. Thus, the type and duration of operation are standardized in this study. Furthermore, the effects of maxillary advancement and impaction, mandibular setback on postoperative pain were evaluated.

It is known that determination of analgesic consumption with PCA is a good indicator in the evaluation of postoperative pain (23). During postoperative 24 hours tramadol was given with PCA to all patients who were included in this study. 24-hour tramadol consumption and VAS were used evaluation of the postoperative pain. It has been reported in the literature that young adults (<25 years old) and women have more opioid analgesic requirements and give higher pain scores after double jaw surgery. In this study, no statistically significant relationship was found between postoperative 24-hour tramadol consumption and VAS values ​​and gender and age variables (*p*> 0.05).

The most common side effects after NSAID use occur on the gastrointestinal tract. It is reported that dexketoprofen has low-to-moderate risk in terms of gastrointestinal bleeding. In this study, no adverse events were observed related to dexketoprofen use in any patient.

Three-dimensional imaging, such as 3D computed tomography (CT) (24) , magnetic resonance imaging (25) , photo and laser surface scanning (12) , are increasingly used to evaluate craniomaxillofacial structures and swelling. The 3dMD facial system (3dMD, Atlanta, GA) is an advanced stereophotogrammetry system that uses multiple cameras to capture a 180° image in just 1.5 ms from the ear of a person's face. Traditional methods have limitations for investigating cranio-maxillofacial changes, but the 3dMD imaging system provides faster, non-invasive and more accurate data stored in digital format. Van der Meer *et al*. (26) determined that 3dMD stereophotogrammetry was accurate and reliable to assess the volume of facial swelling when the volume exceeded 5.9 mL. Semper-hogg *et al*. (27) and Lin *et al*. (28) evaluated the effect of different doses of dexamethasone on edema after orthognathic surgery in 2017 by using 3dMD system. In this study, a 3DMD system was used which allows a more objective and fast evaluation without exposure ionizes radiation compared to other three-dimensional imaging methods. Kau *et al*. (24) reported that approximately 60% of postoperative edema decreased in the first month and the facial morphology was stabilized by approximately 83% at postoperative 3th month. Lin *et al*. (28) reported that edema decreased approximately 86% at the postoperative 1st month. In this study, 3D images were taken at postoperative 90th day (t90) were chosen as the reference to evaluate swelling. There are different opinions about the time of postoperative maximal swelling in the literature. In this study, maximal facial swelling was considered to be approximately 72 hours after surgery. The 3D images were obtained to evaluate swelling postoperative 1th, 3rd, 7th, 14th, 21th, 30th and 90th days. In dexketoprofen group 87.42% of the swelling was resolve and 90.4% of the swelling was resolve in placebo group at the postoperative 1st month.

Precious *et al*. (18) evaluated the effect of PCA iv opioid administration with fixed schedule and dosage, preemptive oral / rectal naproxen and intramusculary codeine on postoperative pain in a study of 75 patients who underwent orthognathic surgery in 1997. They reported that PCA and naproxen group resulted in less opioid requirement than codeine group and high patient satisfaction with low pain scores. The drug administration approach (oral/rectal/intramusculary) and timing of application (preoperative/postoperative) and type of surgeries (Single-double jaw surgery) were not standardized in their study. These factors lead to a lack of reproducibility of this study. Nagatsuka *et al*. (3), performed a smilar study on 82 patients who underwent BSSO in 2001. In the study group, 50mg rectal diclofenac sodium were given, and preoperative bilateral inferior alveolar nerve block was applied 10% butorphanol and 1% lidocaine. They observed lower postoperative VAS scores compared to the placebo. In 2007, Tüzüner *et al*. (7) evaluated the effect of preoperative intramuscular 50 mg tramadol, 75 mg diclofenac sodium and placebo on postoperative pain in 36 patients who underwent double jaw surgery. Tramadol and diclofenac group was provided statistically significantly better pain control compared to placebo with decreased opioid consumption. They stated that there was no statistically significant difference between diclofenac sodium and tramadol. This study was designed prospective, randomized, double blind, controlled to provide the standardization and increase scientific effect of the study. In dexketoprofen group statistically significant decrease was observed in terms of postoperative tramadol consumption compared with placebo in consistent with the literature (*p*=0,015). The mean values of 24-hour tramadol consumption were 109.90 ± 35.54 mg in the dexketoprofen group and 148.17 ± 45.13 mg in the placebo group. Also, postoperative 3rd hour and 24 hours mean VAS values ​​were significantly lower in dexketoprofen group compared to placebo group (*p* <0.001). Although mean VAS values ​​were lower in dexketoprofen group at postoperative 1st hour, there was no statistically significant difference between the two groups. No statistically significant difference was found between the groups in terms of rescue analgesic requirement (*p*> 0.05).

Although there are many studies on the analgesic efficacy of dexketoprofen, studies on anti-inflammatory efficacy are limited. Jimenez *et al*. (29) reported that anti-inflammatory effects of 25 mg dexketoprofen on swelling after impacted third molar surgery was higher than 600 mg ibuprofen. However, in the relevant study, objective criteria were not presented in the evaluation of inflammation. Eroglu *et al*. (25) reported that the anti-inflammatory effects of preemptive dexketoprofen on swelling after third molar surgery was insufficient in two separate studies. Consistent with Eroğlu *et al* (25)., the anti-inflammatory effect of preemptive IV dexketoprofen on swelling after orthognathic surgery was insufficient. There was no statistically significant difference compared to placebo (*p*> 0.05).

The strength of this study is that randomized, clinical and prospective study with objective assessment of pain and swelling, standardization of operation related variables and postoperative care. The limitations of study are absence of comparison with other nonsteroidal agents or without using with combination of local anaesthetics and opioids.

It has been known that the main complaints of the patients after orthognathic surgery are pain and swelling. Currently, there are no fully accepted or approved postoperative analgesia protocols to provide analgesia after orthognathic surgery. For this reason, the concept of “Multimodal Analgesia" is gaining more importance recently. Preventive single dose of iv dexketoprofen administration is an effective and safe method for the management of postoperative pain in patients undergoing double jaw surgery. Future studies are needed to investigate the preemptive effects of different analgesics with larger sample size.
